# Skull-femoral traction after posterior release for correction of adult severe scoliosis: efficacy and complications

**DOI:** 10.1186/s12891-018-2207-3

**Published:** 2018-08-02

**Authors:** Jun Qiao, Lingyan Xiao, Leilei Xu, Zhen Liu, Xu Sun, Bangping Qian, Zezhang Zhu, Yong Qiu

**Affiliations:** 1 Department of Spine Surgery, the Affiliated Drum Tower Hospital of Nanjing University Medical School, 321 Zhongshan Road, Nanjing, China; 2 Intensive care unit, the Second Hospital of Nanjing, Southeast University, Nanjing, China

**Keywords:** Adult severe scoliosis, Skull-femoral traction, Complication

## Abstract

**Background:**

It is a great challenge for spine surgeons to correct severe rigid scoliosis. We developed a three- staged correction (one stage posterior release and screw placement, two stage skull-femoral traction and three stage posterior instrumentation) for adult severe scoliosis. The objective of this study is to investigate safety and efficacy of a three- staged correction for adult severe scoliosis.

**Methods:**

A retrospective review was performed for patients with severe scoliosis receiving three- staged correction (one stage posterior release and screw placement, two stage skull-femoral traction and three stage posterior instrumentation) from June 2001 to October 2014. The inclusion criteria were as follows: [1] age more than 18 years; [2] main curve larger than 90°; [3] a minimum 2-year follow-up. Patients were excluded if they had a history of surgery or anterior release or receiving three column osteotomies.

**Results:**

A total of 63 patients were included (37 female and 26 male), with a mean age of 22.7 years (range: 18–30 years) and follow-up of 42.6 months (range: 24–108 months). The aetiology was congenital in 27 patients, neuromuscular in 18, idiopathic in 11, neurofibromatosis-1 in 4 and Marfan syndrome in 3. The mean traction weight was 28.4 kg (range: 18–32 kg), equal to 57.2% of patients’ body weight (range: 42.7–72.3%). The mean traction time was 22.7 days (range: 12–44 days). Postoperative correction rate was 55% (range: 38–78%) for scoliosis and 51% (range: 32–75%) for kyphosis. Contribution of traction to correction was 51% (range: 36–70%) for scoliosis and was 43% (range: 34–55%) for kyphosis.

**Conclusions:**

Three- staged correction (one stage posterior release and screw placement, two stage skull-femoral traction and three stage posterior instrumentation) could effectively correct adult severe scoliosis. The incidence of complications of skull-femoral traction was not low, but transient and could be successfully managed.

## Background

**Fig. 1:**
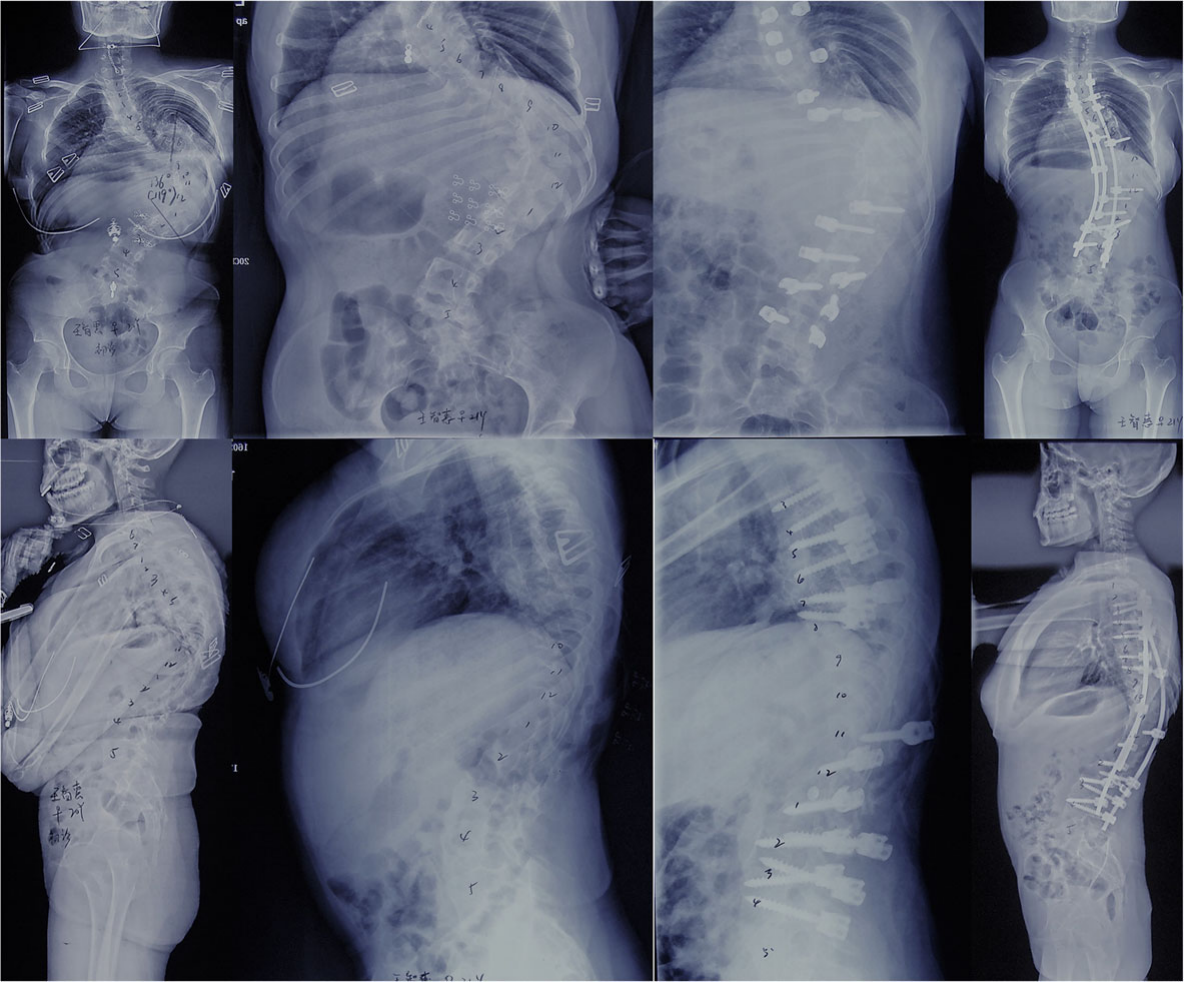
A 21-year-old female patient with congenital kyphoscoliosis had a scoliosis of 136° and a kyphosis of 85°. Major coronal curve decreased to 100° at side bending radiograph, and kyphosis to 75° at supine radiograph. After 3-week traction, scoliosis was corrected to 75°, and kyphosis to 56°. Postoperative standing radiograph demonstrated scoliosis was corrected to 60°, and kyphosis to 40°

It is a great challenge for spine surgeons to correct severe rigid scoliosis and kyphoscoliosis [1]. In addition to large curve, significant pulmonary compromise and neurological deficit would also place patients under risks of surgical complication [2–5]. In the past, multiple forms of traction were employed to increase correction of curve, improve pulmonary function and save neurological function before surgery [6–9]. The development of segmental instrumentation and aggressive osteotomies largely improved correction of severe rigid scoliosis and kyphosis [10–12]. However, traction still has its role in improving pulmonary function and minimizing neurological complications [9, 13, 14]. Moreover, traction could provide wide release of spine that increase flexibility of both primary and secondary curves; whereas, osteotomies could only release a limited range of spine. Halo-gravity traction (HGT) is the most frequently used traction for patients, especially pediatric patients with severe scoliosis or kyphoscoliosis. It could gradually lengthen the height of thoracic spine and rib cage, and enlarge the volume of lungs. Skull-femoral traction was a more aggressive form that simultaneously offer caudal and cephalic traction forces. As compared to HGT, skull-femoral traction (SFT) could generate more traction forces [15, 16]. Moreover, the traction time of SFT is shorter than HGT, mostly less than 4 weeks. SFT is more suitable for adult patients, as most pediatric patients could not bear such big traction forces. In this study, we investigated safety and efficacy of a three- staged correction (one stage posterior release and screw placement, two stage skull-femoral traction and three stage posterior instrumentation) for adult severe scoliosis (Fig. 1).

## Methods

### Patients

A retrospective review was performed for patients with severe scoliosis receiving three- staged correction (one stage posterior release and screw placement, two stage skull-femoral traction and three stage posterior instrumentation) from June 2001 to October 2014. The inclusion criteria were as follows: [1] age more than 18 years; [2] main curve larger than 90°; [3] a minimum 2-year follow-up. Patients were excluded if they had a history of surgery or anterior release or receiving three column osteotomies.

### Radiographic analysis

Standing long-cassette antero-posterior (AP) and lateral radiographs of the whole spine were taken before posterior surgery, 10 days after posterior surgery and at final follow-up respectively. Supine antero-posterior (AP) and lateral radiographs were taken before posterior release and screw placement surgery and under traction before posterior instrumentation. Coronal and sagittal curves were measured by Cobb method. Curve flexibility for scoliosis was initially assessed using the supine side bending films in all patients and calculating the percentage of curve correction on these views. Curve flexibility for kyphosis = (Standing kyphosis- supine kyphosis)/standing kyphosis*100%. Curve correction rate = (initial curve- corrected curve) /initial curve *100%. Contribution of traction to correction = (initial curve-curve after traction)/ (initial curve-postoperative curve) * 100%. CT scans of instrumented levels were performed after first stage surgery to see the positions of pedicle screws.

### Posterior release and screw placement

After a midline incision, subcutaneous tissue and subperiosteal was dissected from the spinous processes, laminae and transverse processes. The supra and interspinous ligaments at each level were completely removed. And then, a complete excision of the ligamentum flavum was performed by means of a Kerrison rongeur. The release of this rongeur was extended throughout the ligament, beginning in the midline and proceeding laterally toward both facets. At last, the inferior facet of the superior vertebra was removed, as was much of the superior facet of the inferior vertebra. This release was performed along the instrumented region at each level. Pedicle screws were then placed by free-hand technique or with the aid of O-arm based navigation system, maintaining the construct density above 70%. Skull-traction was installed after closure of posterior incision.

### Traction protocol

Traction was usually started at the second day after posterior surgery with a weight of 2 kg and gradually increased at a rate of 1 to 2 kg per day if patients well tolerated (Fig.3). The maximum traction weight could be 33 to 50% of the whole body weight depending on patients’ tolerance. Traction was applied for a minimum of 12 h per day, with the traction weight lessened to 50% in the night. If tolerated, traction could be applied for a maximum of 20 h. In the rest of time, they are allowed out of traction for bathroom privileges and hygiene purposes as well as eating. During the traction, the patient’s neurological status was frequently checked. If hyper reflex of the extremities, Babinski sign, paresthesia, dysfunction of cranial nerves or any other neurological compromise were noted, the weight would be immediately reduced. The length of the traction period was mainly determined by the radiographic evidence of curve improvement on weekly radiographs, in addition to clinical evaluation of the patients’ pulmonary and neurological function.

### Posterior instrumentation

Surgery was performed under traction. Pedicle screws were revised if showed mal-placed at postoperative CT scans. Rod was first placed at concave side of the spine, and then convex side. Satellite rods and segmental correction technique were used if necessary. Correction was gradually achieved with a combination of maneuvers including derotation, translation and the application of cantilever forces. A wake-up test was performed and was positive in all patients.

Statistical analysis of the data was performed using SPSS 17.0 software (SPSS Inc., Chicago, IL, USA). Statistical data were presented as the mean ± standard deviation. The changes of radiographic parameters were compared using paired Student’s t test. A Pearson correlation was conducted to assess normally distributed variables. Spearman’s rank correlation method was conducted for nonparametric data. Bivariate analyses for correction rates were conducted first, and variables that were significant at *P* < 0.05 or considered relevant to correction rates from a clinical perspective were entered into multivariable linear regression models. Statistical significance was defined as *P* < 0.05.

## Results

### Patient characteristics

A total of 63 patients were included (37 female and 26 male), with a mean age of 22.7 years (range: 18–30 years) and follow-up of 42.6 months (range: 24–108 months). The aetiology was congenital in 27 patients, neuromuscular in 18, idiopathic in 11, neurofibromatosis-1 in 4 and Marfan syndrome in 3. The mean traction weight was 28.4 kg (range: 18–32 kg), equal to 57.2% of patients’ body weight (range: 42.7–72.3%). The mean traction time was 22.7 days (range: 12–44 days).

### Radiographic analysis

**Fig. 2:**
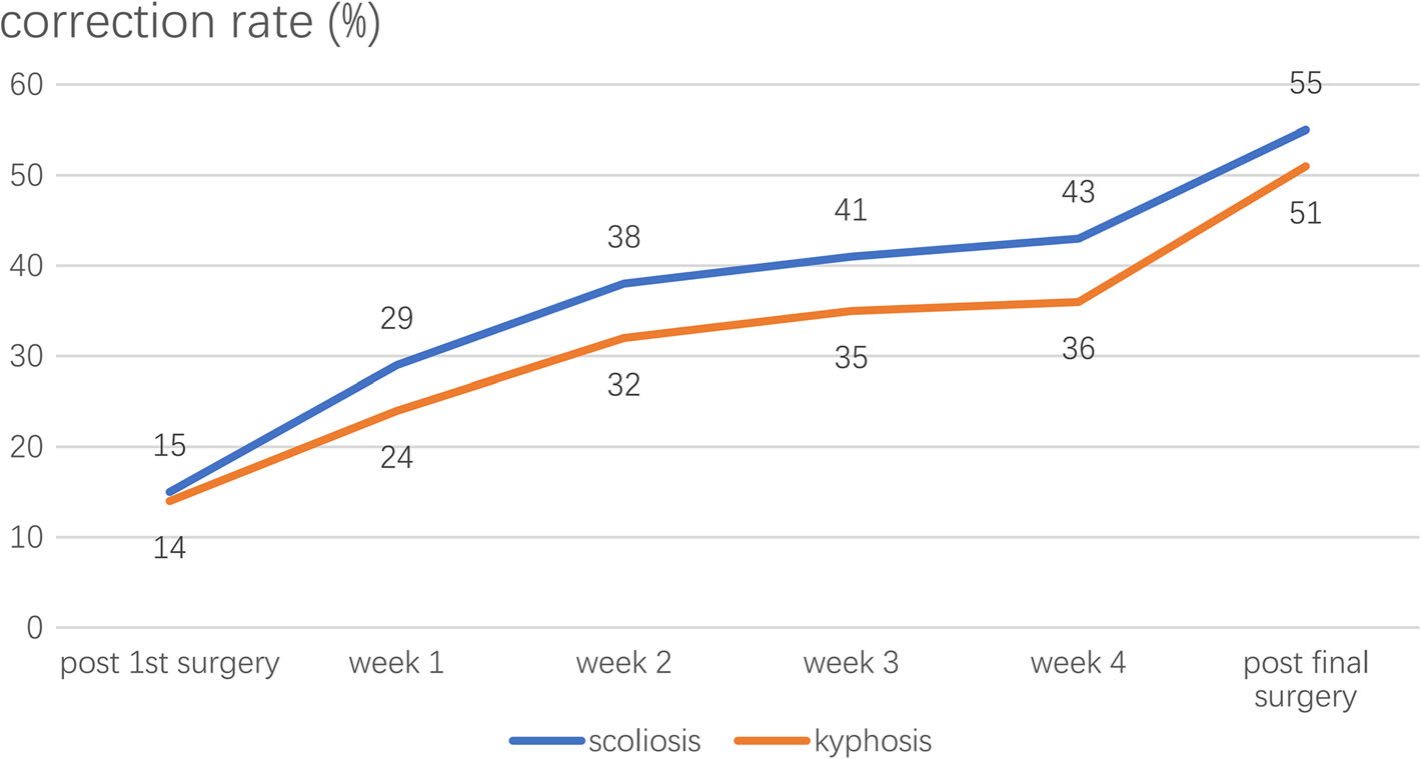
Dynamic changes of correction rates of scoliosis and kyphosis at different stages

The average preoperative coronal Cobb angle of main curve was 118.7° (range: 92° -158°) and was 93.1° (range: 70° -139°) for kyphosis. The mean curve flexibility was 18% (range: 0–37%) for scoliosis and 11% (range: 0–22%) for kyphosis. Both coronal and sagittal were continuously reduced under traction. (Fig. 1) Postoperative correction rate was 55% (range: 38–78%) for scoliosis and 51% (range: 32–75%) for kyphosis. Contribution of traction to correction was 51% (range: 36–70%) for scoliosis and was 43% (range: 34–55%) for kyphosis (Fig. 2). Significant difference of curve severity was noted between initial curve and post-traction curve for both coronal and sagittal deformity (*P* < 0.05). The average postoperative coronal Cobb angle of main curve was 57.3° (range: 29° -96°) and 59.6° (range: 29° -102°) at last follow-up. The average postoperative kyphosis was 46.4° (range: 19°- 76°) and 48.1° (range: 20°-78°) at last follow-up. There is no difference between postoperative curve and follow-up curve for both coronal and sagittal deformity (*P* < 0.05). (Table 1).

**Table 1:** Dynamic changes of curve severity at different stages

	scoliosis	kyphosis
Initial (°)	118.7	93.1
Bending/supine (°)	97.3	83.9
After 1st surgery (°)	99.6	80.1
After traction (°)	67.4	58.2
After final surgery (°)	57.3	46.4
Last follow-up (°)	59.6	48.1

### Factors related to correction rates of traction and correction surgery

Age, preoperative curvature and curve flexibility were correlated to correction rates of traction for scoliosis (age: *r* = − 0.382, preoperative curvature: *r* = − 0.416, curve flexibility: *r* = 0.537; *P* < 0.05). Age and curve flexibility were correlated to correction rates of traction for kyphosis (age: *r* = − 0.405, curve flexibility: *r* = 0.493; P < 0.05). Age, correction rates of traction and curve flexibility were correlated to correction rates of surgical correction for scoliosis (age: *r* = − 0.426, correction rates of traction: *r* = 0.629, curve flexibility: *r* = 0.594; *P* < 0.05). Age, correction rates of traction and curve flexibility were correlated to correction rates of surgical correction for kyphosis (age: *r* = − 0.422, correction rates of traction: *r* = 0.568, curve flexibility: *r* = 0.483; *P* < 0.05). (Table 2).

**Table 2:** Factors related to correction rates of traction and correction surgery

	Scoliosis traction rate		Kyphosis traction rate		Scoliosis correction rate		Kyphosis correction rate	
	*r*	*p*	*r*	*p*	*r*	*p*	*r*	*p*
Age	−0.382	0.032^a^	− 0.405	0.027^a^	− 0.426	0.009^a^	− 0.422	0.018^a^
Preoperative scoliosis	−0.416	0.017^a^	–	–	0.218	0.104	–	–
Scoliosis flexibility	0.537	0.006^a^	–	–	0.594	0.002*	–	–
Preoperative kyohisis	–	–	0.176	0.094	–	–	0.228	0.058
Kyphosis flexibility	–	–	0.493	0.017^a^	–	–	0.483	0.021^a^
Scoliosis traction rate	–	–	–	–	0.629	0.002^a^	–	–
Kyphosis traction rate	–	–	–	–			0.568	0.003^a^

^a^:*p* < 0.05

Multivariable regression analyses found that curve flexibility was related to correction rates of traction for both scoliosis (r^2^ = 0.392; P < 0.05) and kyphosis (r^2^ = 0.375; P < 0.05). Correction rates of traction (r^2^ = 0.421; P < 0.05) and curve flexibility (r^2^ = 0.316; P < 0.05) were related to correction rates of surgery for scoliosis. Correction rates of traction (r^2^ = 0.489; P < 0.05) and curve flexibility (r^2^ = 0.292; *P* < 0.05) were related to correction rates of surgery for kyphosis.

### Complications

#### Surgical complications

In first stage surgery, 17 misplaced screws were observed at 12 patients, and were revised at final surgery. 2 patients developed pleural rupture during last surgery and were sutured operatively. One of the two patients with pleural rupture developed pleural effusion and underwent thoracic close drainage. Neurological deficit was observed at one patient after last surgery. For this patient, the muscle strength of left leg decreased to grade 4 after surgery, and completely recovered at final follow-up. Totally, the incidence of surgical complications was 19.0% for first stage surgery, and 4.8% for final surgery.

#### Traction complications

Two patients suffered from brachial plexus palsy and one patient femoral nerve palsy. However, complete nerve function restoration was achieved at final follow-up. Transient hematuria occurred in two patients. Gastrointestinal symptoms developed in one patient, and after reducing traction weight, the symptoms relived. Deep vein thrombosis (DVT) developed in two patients, and one patient underwent inferior vena filter placement. Pin infection occurred in two patients, and was controlled by debridement. Totally, the incidence of traction complications was 11.1%.

## Discussion

Several forms of traction were used for correcting severe scoliosis [2, 8, 15, 17]. Halo-gravity traction (HGT) was the most frequently used traction by using the weight of the body as a counterforce. It can be applied while a patient is in bed or on a wheelchair; thus, allowing patients to be out of bed to socialize and participate in exercise programs [18]. The most significant disadvantage of HGT may be the prolonged hospital stay, which was not accepted by all families. The effect of HGT on curve correction was controversial. Most studies did not support that preoperative-traction would provide superior curve correction to immediate spinal fusion with instrumentation. Seller [19] investigated efficacy of HGT on correction of neuromuscular scoliosis, and found that the surgical correction rates did not differ with or without preop-HGT. Sponseller [20] compared surgical correction of severe spine deformity with preoperative halo traction and without preoperative traction, and found there was no statistically significant difference in main coronal curve correction (62% vs. 59%), operative time, blood loss, and total complication rate (27% vs. 52%) between the two groups. Koller [13] also claimed that HGT should not be expected to significantly improve severe curves without a prior anterior and/or posterior release. However, as compared to increasing curve correction, pulmonary function improvement and recovery of neurological deficit achieved by HGT seems more important. Although no additional curve correction was achieved by HGT, Koller [13] demonstrated that FVC% was improved from 42 ± 20 (14–98) % to 49 ± 20 (19–100) % after HGT in patients with severe and rigid scoliosis and kyphoscoliosis. In their subgroup analysis, five patients with kyphoscoliosis and progressive neurological deficits from a decompensating curve showed improvement after the initiation of HGT. HGT could lead to a slight curve correction and then causes a release of the apical tether on the spinal cord.

Some additional procedures were administrated in combination with traction to boost clinical and radiographic outcomes. Park [21] used an anterior release- HGT- posterior instrumentation protocol to correct severe pediatric spinal deformity getting 66.3% of major coronal curve correction and 62.7% of sagittal curve correction. Koptan [8] compared a three-staged correction by an anterior release, 2 weeks of halo-gravity traction then posterior instrumentation (TRN group) with a two-staged correction by anterior release then posterior instrumentation (SAP group), and found that the application of gradual traction over a limited period of 2 weeks in addition to anterior release led to better correction. Bao [22] reported a group of adult scoliosis patients with respiratory dysfunction undergoing HGT combined with assisted ventilation, and demonstrated that combined HGT and assisted ventilation would be beneficial to pulmonary function improvement in severe adult scoliosis cases. Halo-femoral traction was also popular in correcting severe scoliosis. Qiu [6] used an anterior release- halo femoral traction- posterior instrumentation to correct severe idiopathic and congenital scoliosis, and got a 57.5%. correction of major curve for idiopathic scoliosis and 45.2% for congenital scoliosis. Wang [23] used preoperative halo-femoral plus posterior vertebral column resection to correct extremely severe rigid spinal deformity with sharp angular curve > 150°, and achieved 69% correction of scoliosis and 66% correction of kyphosis. The present protocol was applied for the patients with good tolerance of heavy traction and without neurological compromise. If a patient had neurological compromise, we preferred a halo-gravity traction before correction surgery. The advantages of this protocol could be three folds: first, a long and difficult correction surgery for severe scoliosis was divided to two phases minimizing surgical complications and providing intermittent recovery period for patients; second, posterior release had less complications and pulmonary compromise as compared to anterior release; third, heavy bi-directional traction by skull-femoral traction provided more traction forces and entailed less traction time as compared to HGT. In our cohort, correction rate of scoliosis reached 55% and kyphosis 58%. The contribution of traction to correction was 51% for scoliosis and 43% for kyphosis. As other forms of traction, skull-femoral traction after posterior release attained most of correction within 2 weeks, and after 3 weeks, the traction corrected deformity much slower than the first 2 weeks. 3 weeks of traction is enough for most patients.

Prevalence of traction-related complications ranged from 16 to 28% [24]. Pin-related complications ranked first, including pin loosening and superficial pin-site infection. In our study, pin-related complications occurred at 2 patients, both of which were infection. After debridement, infection was successfully controlled. The most concerning complication was neurological deficit caused by heavy weight traction [14, 25]. In our hospital, skull- femoral traction was always prescribed to adult patients, because they have better tolerance as compared to pediatric patients. However, there were still two cases of brachial plexus palsy and one case of femoral nerve palsy. After reducing traction weight, symptoms relived in all three patients. Deep vein thrombosis (DVT) may be a unique complication for skull-femoral traction, because the patients were immobilized during traction [26]. To avoid this complication, we recommended the use of anticoagulant therapy during traction [27]. Other complications included two cases of transient hematuria and one case of gastrointestinal symptoms, which were relieved by reducing traction weight. Generally, the incidence of complications of skull-femoral traction was not low, but transient and could be successfully managed.

Seventeen misplaced screws were observed at 12 patients at CT scans. At final surgery, we revised the positions of these screws. For some severe scoliosis, especially scoliosis associated with Marfan syndrome and neurofibromatosis-1, pedicles were extremely thin in concave side of apex region, and misplacement would be inevitable by free-hand technique. Staged correction protocol provides opportunity to revise misplaced screws that avoids neurological compromise and correction loss caused by misplaced screws. Only one case of incomplete neurological deficit occurred, and finally recovered. Traction may increase the tolerance of spinal cord to stretch trees and ischemia from correction of curve, diminishing risks of neurological complications.

The most significant limitation of this three-staged correction procedure was its inability to manage patients with pulmonary compromise because posterior release and screw placement should be performed prior to traction. A patient with pulmonary compromise may not tolerate fist stage surgery. In addition, bidirectional heavy weight traction was not suitable for pediatric patients in consideration of their poor tolerance. Finally, back pain and joint stiffness should also draw attention.

Another limitation of this study was unavailability of psychological status of patients receiving this complex treatment. Long time bed-bound heavy traction would pose significant mental stress on patients. Further study is needed to evaluate psychological status of these patients by using reliable questionnaires. In addition, heterogeneity of etiology was also a limitation. However, correlation analysis did not find correlation between etiology and surgical outcomes.

## Conclusion

In conclusion, three- staged correction (one stage posterior release and screw placement, two stage skull-femoral traction and three stage posterior instrumentation) could effectively correct adult severe scoliosis. The incidence of complications of skull-femoral traction was not low, but transient and could be successfully managed.

## Abbreviations

HGT: Halo-gravity traction
SFT: Skull-femoral traction
